# A case with IGG4-related retroperitoneal fibrosis-periaortitis rapidly diagnosed and dramatically responded to steroid treatment

**DOI:** 10.1186/1546-0096-13-S1-P2

**Published:** 2015-09-28

**Authors:** Y Karaaslan, Z Ozbalkan Aslar, S Can Sandikci

**Affiliations:** 1 Hitit University Medical Faculty, Rheumatology, Çorum, Turkey; 2 Ankara Numune Education and Research Hospital, Rheumatology, Ankara, Turkey

## Background

Retroperitoneal fibrosis is a rare disease characterized by development of fibro-inflammatory tissue, which surrounds and causes compression of the retroperitoneal structures such as abdominal aorta, iliac vessels, vena cava and ureters^1^. It's prevalence was reported as 1.4 /100,000 ^2^. It has been recently shown that it is one of the IgG4-related disorders^3^. Herein, we report a male patient who admitted to emergency department with acute abdominal pain, diagnosed with IgG4-related retroperitoneal fibrosis-periaortitis in a very short time, and both high baseline serum creatinine level as well as abdominal pain requiring opioids on admission improved with steroids.

## Case

Forth-one-year-old male admitted to emergency department with the complaint of abdominal pain. Wall of abdominal aorta seemed thickened on ultrasonography (USG). Abdominal computerized tomographic angiography showed soft tissues surrounding aorta, beginning 9 cmproximal to iliac bifurcation and continuing up to the level of common iliac artery (aortitis? retroperitoneal fibrosis?), and the patient was referred to rheumatology clinic. The patient was hospitalized for further investigations. He did not complain of fever, fatigue, or urinary symptoms. The blood tests revealed the following: Erythrocyte sedimentation rate (ESR) 57 mm/h, CRP 43 mg/L, HGB 14.1 g/dL, WBC count 9800/μL, PLT count 306000/μL, MCV 82.3 fL, creatinine 1.15 mg/dL and ALT 18 U/L. The urinalysis of the patients was normal, and he was negative for ANA, ANCA and ENA. His IgM was 128 mg/dL (46-304), IgG was 2060 mg/dL (751-1560), IgA was 415 mg/dL (82-453), IgG_1_ was 10700 mg/L (3824-9286), IgG_2_ was 9070 mg/L (2418-7003), IgG_3_ was 1400 mg/L (218-1761) and IgG_4_ was 3550 mg/L (39.2-864). IgG_4_/total IgG ratio was 17.2%. Aortoiliac arterial Doppler USG showed that the soft tissue surrounded the left ureter completely and the right ureter partially, and it caused grade 1 hydronephrosis and obstructive volume increase in the left kidney. The patient was diagnosed with IgG4 related disease and retroperitoneal fibrosis, and administered 60 mg methyl prednisolone as well as amlodipine due to high blood pressure. Venous and colored arterial Doppler examinations of the lower extremities were normal. The patient needed narcotic analgesics due to severe abdominal pain at the time of hospitalization. His abdominal pain improved with steroids in one week, and his ESR, CRP and creatinine levels decreased. ESR was 7 mm/h, and serum creatinine was 0.84 mg/dL at the second week of the treatment. The patient was discharged from the hospital with steroids, amlodipine, and PPI, and called for follow up visit 2 weeks later.

We did not biopsy the lesion since history, radiological findings and blood tests were characteristic for IgG_4_ mediated retroperitoneal fibrosis-periaortitis. Most of the cases with IgG_4_ mediated retroperitoneal fibrosis reported in the literature had a long diagnostic delay. Our patient is interesting since he was diagnosed with the disease shortly after beginning of his symptoms, steroids were administered immediately, and he responded steroids dramatically.

## Consent to publish

Written informated consent for publication of their clinical details was obtained from the patient/parent/guardian/relative of the patient.
